# The Effect of Chronic Kidney Disease on Adverse In‐Hospital Outcomes at Radical Prostatectomy

**DOI:** 10.1111/iju.70038

**Published:** 2025-03-14

**Authors:** Fabian Falkenbach, Natali Rodriguez Peñaranda, Mattia Longoni, Andrea Marmiroli, Quynh Chi Le, Calogero Catanzaro, Michele Nicolazzini, Zhe Tian, Jordan A. Goyal, Stefano Puliatti, Riccardo Schiavina, Carlotta Palumbo, Gennaro Musi, Felix K. H. Chun, Alberto Briganti, Fred Saad, Shahrokh F. Shariat, Lars Budäus, Markus Graefen, Pierre I. Karakiewicz

**Affiliations:** ^1^ Cancer Prognostics and Health Outcomes Unit, Division of Urology University of Montréal Health Center Montréal Québec Canada; ^2^ Martini‐Klinik Prostate Cancer Center University Medical Center Hamburg‐Eppendorf Hamburg Germany; ^3^ Department of Urology Ospedale Policlinico e Nuovo Ospedale Civile S. Agostino Estense Modena, University of Modena and Reggio Emilia Modena Italy; ^4^ Vita‐Salute San Raffaele University Milan Italy; ^5^ Department of Urology IEO European Institute of Oncology, IRCCS Milan Italy; ^6^ Department of Oncology and Haemato‐Oncology Università degli Studi di Milano Milan Italy; ^7^ Department of Urology Goethe University Frankfurt, University Hospital Frankfurt am Main Germany; ^8^ Division of Urology IRCCS Azienda Ospedaliero‐universitaria di Bologna Bologna Italy; ^9^ Division of Urology, Department of Translational Medicine University of Eastern Piedmont, Maggiore della Carità Hospital Novara Italy; ^10^ Division of Urology, Department of Oncology University of Turin Orbassano Italy; ^11^ Department of Urology Comprehensive Cancer Center, Medical University of Vienna Vienna Austria; ^12^ Department of Urology Weill Cornell Medical College New York New York USA; ^13^ Department of Urology University of Texas Southwestern Medical Center Dallas Texas USA; ^14^ Hourani Center for Applied Scientific Research Al‐Ahliyya Amman University Amman Jordan; ^15^ Department of Urology University Medical Center Hamburg‐Eppendorf Hamburg Germany

**Keywords:** chronic kidney disease, NIS, prostate cancer, prostatectomy

## Abstract

**Objective:**

Radical prostatectomy (RP) may be a treatment option for prostate cancer in patients with chronic kidney disease (CKD). However, the effect of CKD on adverse in‐hospital outcomes after RP is not well known.

**Methods:**

Descriptive analyses, propensity score matching (PSM), and multivariable logistic and Poisson regression models were used to address National Inpatient Sample RP patients between 2005 and 2019. CKD severity was stratified as mild (stage I/II) versus moderate (stage III) versus severe (stage IV/V).

**Results:**

Of 191 050 RP patients, 4349 (2.3%) had CKD. Of those, 2301 (52.9%), 1416 (32.6%), and 632 (14.5%) were classified as mild, moderate, or severe CKD, respectively. The CKD rate increased from 0.3% to 5.6% (2005–2019, EAPC: + 15.3%, *p* < 0.001). CKD patients invariably exhibited higher rates of adverse in‐hospital outcomes, except for in‐hospital mortality. The absolute differences were largest for overall complications (+ 12.5%), length of stay > 2 days (+ 11.8%), and blood transfusions (+ 3.7%, all *p* < 0.001). CKD was an independent predictor in all comparisons except for in‐hospital mortality (*p* < 0.05). The detrimental effect was most pronounced for dialysis for acute kidney failure (multivariable odds ratio [OR] 10.49), genitourinary complications (OR: 2.47), and critical care therapies (OR: 2.45, all *p* < 0.001). Finally, a dose–response relationship of CKD severity (mild vs. moderate vs. severe) and its effect on adverse in‐hospital outcomes was observed in seven of 14 comparisons.

**Conclusions:**

CKD patients invariably exhibited higher rates of adverse in‐hospital outcomes after RP. The presence of CKD should be carefully considered when RP represents a management option.

## 1 Introduction

Radical prostatectomy (RP) may represent a treatment option for patients with chronic kidney disease (CKD) and prostate cancer (PCa) [1, 2]. CKD has been independently associated with higher rates of complications and poor outcomes after several different surgical procedures, including RP [3, 4, 5, 6]. In detail, two previous reports examined adverse outcomes after RP in CKD patients. Schmid et al. reported on the effect of CKD on 30‐day outcomes in major urological oncological surgeries between 2005 and 2011, which also included RP patients [4]. Ning et al. reported on adverse in‐hospital outcomes in RP patients with underlying CKD between 2005 and 2014 [5]. Since their publication, the management and consideration of various baseline comorbidities at RP, including CKD, may have changed. Consequently, we hypothesized that CKD patients may no longer be at a significant disadvantage when adverse in‐hospital outcomes after RP are considered. We tested this hypothesis in a contemporary, population‐based, large‐scale cohort of RP patients within the National Inpatient Sample (NIS) from 2005 to 2019.

## 2 Materials and Methods

### 2.1 Data Source

Our analysis relied on hospital discharge data from the NIS (2005–2019) to address adverse in‐hospital outcomes after RP. The longitudinal NIS databases are included in the Healthcare Cost and Utilization Project (HCUP) by the Agency for Healthcare Research and Quality (AHRQ) within a Federal‐State partnership [7]. The International Classification of Disease (ICD) 9th revision Clinical Modification (ICD‐9‐CM), ICD 10th revision Clinical Modification (ICD‐10‐CM), and the ICD 10th revision Procedure Coding System (ICD‐10‐PCS) were applied to identify diseases, outcomes, and procedures.

### 2.2 Study Population

We focused on patients with a primary diagnosis of PCa (ICD‐9‐CM codes 185, 233.4, 236.5, V10.46; ICD‐10‐CM codes C61, D07.5, D40.0, Z85.46). Only patients treated with RP were included, in accordance with the previously established methodology [8, 9]. Patients were stratified according to the underlying diagnosis of chronic kidney disease (ICD‐9‐CM code 585; ICD‐10‐CM code N18). As each patient can undergo RP only once, each captured hospital encounter in the NIS represents one distinct patient.

### 2.3 Outcomes of Interest

Our primary endpoints consisted of adverse in‐hospital outcomes, defined as overall complications, rates of critical care therapy (without dialysis), rates of dialysis for acute kidney failure, bleeding complications, rates of blood transfusions, cardiac complications, respiratory complications, genitourinary complications, wound complications, infectious complications, and vascular complications, identified by ICD‐9 and ICD‐10 codes according to previously reported methodology [10, 11]. Moreover, we assessed in‐hospital mortality, prolonged length of stay (LOS, > 2 days), and total hospital charges (THC). THC were obtained directly from the NIS and were based on accounting reports in accordance with the NIS methodological guidelines [7]. Within the NIS, THC is adjusted to the 2016 United States dollar (US$), and their definition relies on the overall Consumer Price index by the US Bureau of Labor Statistics [12]. The Deyo modification of the Charlson's Comorbidity Index (CCI) was applied to account for comorbidities [13]. The point contribution to CCI due to CKD was subtracted to compare additional comorbidities besides their renal condition. We relied on the coding algorithms for defining comorbidities using ICD‐9‐CM and ICD‐10‐CM codes by Quan et al. [14]. The covariables included age (years, continuously coded), ethnicity (Caucasian vs. Afro‐American vs. others), adjusted CCI (0 vs. 1 vs. ≥ 2), robot‐assisted approach (yes vs. no), pelvic lymph node dissection (PLND; yes vs. no), year of surgery (2009–2014 vs. 2015–2019), and hospital size (large vs. others). CKD severity was classified as mild (stage I/II, equivalent to a glomerular filtration rate (GFR) ≥ 60 mL/min/1.73/m^2^ or unspecified), moderate (stage III, equivalent to a GFR 30–59 mL/min/1.73/m^2^), and severe/end‐stage (stage IV/V, equivalent to a GFR < 30 mL/min/1.73/m^2^), identified by ICD‐9 and ICD‐10 codes.

Additional sensitivity analyses verified our findings in the subgroup of robot‐assisted RP patients (Tables S1–S3).

### 2.4 Statistical Analyses

First, patient, hospital, and surgery characteristics, as well as adverse in‐hospital outcomes, were tabulated. For continuously coded variables, medians and interquartile ranges (IQR) were calculated. For categorical coded variables, frequencies and proportions were calculated. Wilcoxon rank‐sum and Pearson´s chi‐squared tests were used to test for differences between CKD and non‐CKD patients. Second, the estimated annual percentage changes (EAPC) for the proportion of CKD (overall/stage‐specific) within all RP patients were calculated using least‐squares linear regression. Third, we performed 1:3 propensity score matching (PSM) between CKD and non‐CKD patients. PSM relied on age, ethnicity, adjusted CCI, surgical approach, PLND, year of surgery, and hospital size, according to the nearest neighbor. The objective of PSM was to maximally reduce and ideally eliminate the effects of bias and confounding. Fourth, multivariable logistic and Poisson regression models predicting adverse in‐hospital outcomes were fitted after adjusting for clustering at the hospital level using a generalized estimation equation methodology [11]. The analyses followed the NIS reporting guidelines, and counts were censored for samples < 11 [7]. R software was used for statistical computing and graphics (R version 4.3.1 (2023‐06‐16); The R Foundation for Statistical Computing, Vienna, Austria). All tests were two‐sided, with a significance level set at *p* < 0.05.

## 3 Results

### 3.1 Overall Characteristics of the Study Population

**FIGURE 1:**
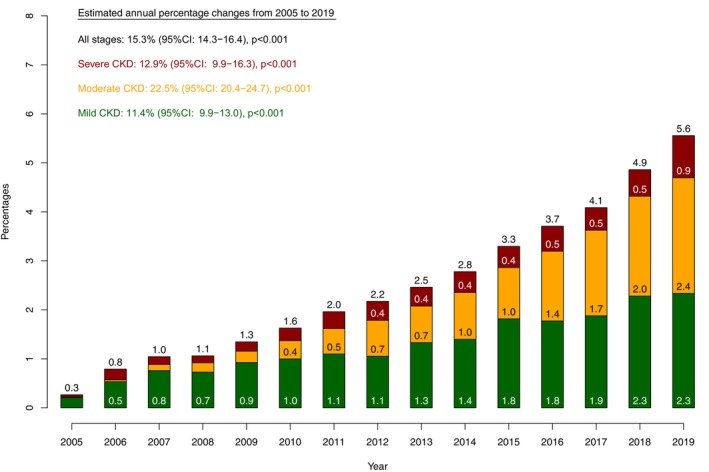
Proportion of prostate cancer patients undergoing radical prostatectomy with chronic kidney disease (all stages/stage‐specific) over time within the National Inpatient Sample (NIS) from 2005 to 2019.

Of the 191 050 patients who underwent RP for localized PCa between 2005 and 2019, CKD was reported in 4349 (2.3%). Of these, 2301 (52.9%), 1416 (32.6%), and 632 (14.5%) patients were classified as mild, moderate, and severe CKD, respectively (Table 1). The proportion of CKD within all RP patients increased from 0.3% in 2005 to 5.6% in 2019 (EAPC: +15.3%, *p* < 0.001, Figure 1). In detail, the percentage of mild, moderate, and severe CKD at RP increased from 0.2% to 2.3% (EAPC: +11.4%), from 0.01% to 2.4% (EAPC: +22.5%), and from 0.05% to 0.9% (EAPC: +12.9%; all *p* < 0.001), respectively.

**TABLE 1:** Descriptive characteristics of prostate cancer patients undergoing radical prostatectomy, stratified according to presence or absence of chronic kidney disease, prior and after 1:3 propensity score matching (PSM).

Characteristic	Prior PSM			After 1:3 PSM		
	With CKD (*n* = 4349, 2.3%)	Without CKD (*n* = 186 701, 97.7%)	*p* ^a^	With CKD (*n* = 4349, 25.0%)	Without CKD (*n* = 13 047, 75.0%)	*p* ^a^
Age, median (IQR), in years	66 (61, 70)	62 (57, 67)	< 0.001	66 (61, 70)	66 (61, 70)	0.5
CKD stage, *n* (%)			NA			NA
Mild (stage I/II)	2301 (52.9%)	0 (0%)		2301 (52.9%)	0 (0%)	
Moderate (stage III)	1416 (32.6%)	0 (0%)		1416 (32.6%)	0 (0%)	
Severe/end‐stage (stage IV/V)	632 (14.5%)	0 (0%)		632 (14.5%)	0 (0%)	
Adjusted CCI^b^, *n* (%)			< 0.001			0.7
0	1854 (42.6%)	139 347 (74.6%)		1854 (42.6%)	5576 (42.7%)	
1	1022 (23.5%)	36 280 (19.4%)		1022 (23.5%)	3126 (24.0%)	
≥ 2	1473 (33.9%)	11 074 (5.9%)		1473 (33.9%)	4345 (33.3%)	
Ethnicity, *n* (%)			< 0.001			0.1
Caucasians	2346 (53.9%)	122 547 (65.6%)		2346 (53.9%)	7249 (55.6%)	
African‐Americans	1126 (25.9%)	20 169 (10.8%)		1126 (25.9%)	3318 (25.4%)	
Others	877 (20.2%)	43 985 (23.6%)		877 (20.2%)	2480 (19.0%)	
Robot‐assisted approach, *n* (%)	2791 (64.2%)	100 083 (53.6%)	< 0.001	2791 (64.2%)	8504 (65.2%)	0.2
PLND, *n* (%)	2209 (50.8%)	87 335 (46.8%)	< 0.001	2209 (50.8%)	6594 (50.5%)	0.8
Year of surgery, *n* (%)	2015 (2011, 2017)	2011 (2008, 2015)	< 0.001	2015 (2011, 2017)	2015 (2011, 2017)	0.6
Small‐/medium‐sized hospital, *n* (%)	1631 (37.5%)	64 913 (34.8%)	< 0.001	1631 (37.5%)	4872 (37.3%)	0.9

*Note:* PSM relied on patient age, ethnicity, Charlson's Comorbidity Index (adjusted), surgical approach, PLND, year of surgery, and hospital size.

Abbreviations: CKD = chronic kidney disease, IQR = interquartile range, PLND = pelvic lymph node dissection, PSM = propensity score matching.

^a^ Wilcoxon rank‐sum test, Pearson's chi‐squared test.

^b^ The point contribution to CCI due to CKD was subtracted.

Before PSM, CKD patients were older (66 vs. 62 years), more frequently African American (25.9% vs. 10.8%), and exhibited a higher rate of CCI ≥ 2 (33.9% vs. 5.9%). Moreover, CKD patients more frequently underwent robot‐assisted RP (64.2% vs. 53.6%) and more frequently underwent concomitant PLND (50.8% vs. 46.8%). In CKD patients, a larger proportion of RP was recorded in recent years (median, 2015 vs. 2011). CKD patients were more frequently admitted to small‐/medium‐sized hospitals for RP (37.5% vs. 34.8%, all *p* < 0.001).

After 1:3 PSM, 4349 of 4349 (100%) CKD patients and 13 047 of 186 701 (7.0%) non‐CKD patients remained for further analysis. After PSM, comparisons addressing patient (age, ethnicity, and CCI), hospital (size), and surgery (approach, PLND, and year of surgery) characteristics did not reveal any residual differences (Table 1).

### 3.2 Chronic Kidney Disease Versus Adverse In‐Hospital Outcomes

Compared with their non‐CKD counterparts, CKD RP patients exhibited higher rates of adverse in‐hospital outcomes in all examined categories, except for in‐hospital mortality (all *p* < 0.05; Table 2). Specifically, CKD was associated with higher rates of overall complications (32.8% vs. 20.3%, Δ 12.5%), critical care therapies (2.5% vs. 1.0%, Δ 1.5%), dialyses for acute kidney failure (0.7% vs. < 0.1%, Δ > 0.6%), bleeding complications (1.8% vs. 1.1%, Δ 0.7%), blood transfusions (8.0% vs. 4.3%, Δ 3.7%), cardiac complications (8.3% vs. 5.5%, Δ 2.8%), respiratory complications (4.7% vs. 2.1%, Δ 2.6%), genitourinary complications (5.1% vs. 2.1%, Δ 3.0%), wound complications (0.5% vs. 0.3%, Δ 0.2%), infectious complications (1.8% vs. 0.8%, Δ 0.9%), vascular complications (1.4% vs. 0.9%, Δ 0.5%), in‐hospital mortality (0.3% vs. 0.2%, Δ 0.1%), and LOS > 2 days (37.9% vs. 26.1%, Δ 11.8%). Moreover, the median THC was 5640 US$ higher in CKD patients relative to their non‐CKD counterparts (54 160 vs. 48 520 US$, *p* < 0.001).

**TABLE 2:** Adverse in‐hospital outcomes after radical prostatectomy for localized prostate cancer, stratified according to the presence or absence of chronic kidney disease after 1:3 propensity score matching (*n* = 17 396).

Characteristics	With CKD (*n* = 4349, 25.0%)	Without CKD (*n* = 13 047, 75.0%)	Difference (Δ)	*p* ^a^
Overall complications, *n* (%)	1427 (32.8%)	2655 (20.3%)	12.5%	< 0.001
Critical care therapies (without dialysis), *n* (%)	107 (2.5%)	131 (1.0%)	1.5%	< 0.001
Dialysis for acute kidney failure, *n* (%)	31 (0.7%)	< 11 (< 0.1%)	> 0.6%	< 0.001
Bleeding complications, *n* (%)	77 (1.8%)	143 (1.1%)	0.7%	< 0.001
Blood transfusions, *n* (%)	350 (8.0%)	559 (4.3%)	3.7%	< 0.001
Cardiac complications, *n* (%)	360 (8.3%)	715 (5.5%)	2.8%	< 0.001
Respiratory complications, *n* (%)	206 (4.7%)	268 (2.1%)	2.6%	< 0.001
Genitourinary complications, *n* (%)	221 (5.1%)	276 (2.1%)	3.0%	< 0.001
Wound complications, *n* (%)	23 (0.5%)	40 (0.3%)	0.2%	0.049
Infectious complications, *n* (%)	77 (1.8%)	110 (0.8%)	0.9%	< 0.001
Vascular complications, *n* (%)	62 (1.4%)	121 (0.9%)	0.5%	0.007
In‐hospital mortality, *n* (%)	13 (0.3%)	23 (0.2%)	0.1%	0.2
Prolonged LOS (> 2 days)^b^, *n* (%)	1650 (37.9%)	3399 (26.1%)	11.8%	< 0.001
THC, median (IQR), in US$	54 160 (36 570, 86 540)	48 520 (33 210, 73 370)	5640 US$	< 0.001

Abbreviations: CKD = chronic kidney disease, IQR = interquartile range, LOS = length of stay, THC = total hospital charges.

^a^ Wilcoxon rank‐sum test and Pearson's chi‐squared test.

^b^ Exceeds the third quartile for the cohort.

After full multivariable adjustment for patient (age, ethnicity, and CCI), hospital (large size), and surgery (approach, PLND, and year of surgery) characteristics, CKD independently predicted higher rates of adverse in‐hospital outcomes in all examined categories, except for in‐hospital mortality (all *p* < 0.05, Table 3). Specifically, CKD independently predicted higher overall complications (multivariable odds ratio [OR] 1.98), critical care therapies (OR 2.45), dialysis rates for acute kidney failure (OR 10.49), bleeding complications (OR 1.63), blood transfusions (OR 1.95), cardiac complications (OR 1.58), respiratory complications (OR 2.33), genitourinary complications (OR 2.47), wound complications (OR 1.70), infectious complications (OR 2.01), vascular complications (OR 1.51), LOS > 2 days (OR 1.77), and THC (incidence rate ratio (IRR) 1.18).

**TABLE 3:** Multivariable regression models predicting adverse in‐hospital outcomes according to the presence or absence of chronic kidney disease (overall, stage‐specific) at radical prostatectomy, after 1:3 PSM and adjustment for clustering at the hospital level using generalized estimation equation methodology (*n* = 17 396).

Characteristic	Multivariable OR/IRR (95% CI)			
	CKD (all stages, *n* = 4349)	Mild CKD (stage I/II, *n* = 2301)	Moderate CKD (stage III, *n* = 1416)	Severe/End‐stage CKD (stage IV/V, *n* = 632)
Overall complications	1.98 (1.82–2.15)***	1.83 (1.65–2.03)***	2.04 (1.80–2.32)***	2.41 (2.02–2.88)***
Critical care therapies (without dialysis)	2.45 (1.88–3.19)***	1.86 (1.31–2.63)***	2.95 (2.05–4.23)***	3.37 (2.08–5.44)***
Dialysis for acute kidney failure	10.49 (4.97–22.11)***	2.95 (1.01–8.65)*	10.25 (3.92–26.77)***	42.77 (18.32–99.84)***
Bleeding complications	1.63 (1.24–2.14)***	1.64 (1.17–2.30)**	1.09 (0.64–1.86)	2.60 (1.60–4.23)***
Blood transfusions	1.95 (1.69–2.25)***	1.66 (1.38–1.98)***	1.72 (1.37–2.16)***	3.69 (2.85–4.77)***
Cardiac complications	1.58 (1.37–1.82)***	1.21 (0.98–1.48)	1.92 (1.59–2.32)***	1.97 (1.42–2.73)***
Respiratory complications	2.33 (1.93–2.81)***	2.03 (1.59–2.58)***	2.93 (2.26–3.78)***	2.08 (1.36–3.18)***
Genitourinary complications	2.47 (2.05–2.97)***	2.46 (1.95–3.11)***	2.44 (1.86–3.19)***	2.56 (1.74–3.76)***
Wound complications	1.70 (1.01–2.84)*	1.73 (0.91–3.31)	1.74 (0.79–3.82)	1.48 (0.45–4.89)
Infectious complications	2.01 (1.48–2.74)***	1.67 (1.09–2.55)*	2.33 (1.55–3.52)***	2.20 (1.16–4.17)*
Vascular complications	1.51 (1.10–2.08)*	1.67 (1.13–2.47)*	1.46 (0.93–2.30)	1.11 (0.42–2.88)
In‐hospital mortality	1.61 (0.80–3.22)	1.25 (0.46–3.37)	1.61 (0.58–4.49)	3.06 (0.90–10.41)
Prolonged LOS (> 2 days)^a^	1.77 (1.64–1.91)***	1.55 (1.40–1.71)***	1.78 (1.58–2.01)***	2.77 (2.32–3.32)***
Total hospital charges	1.18 (1.14–1.21)***	1.13 (1.09–1.17)**	1.21 (1.15–1.28)**	1.25 (1.16–1.35)**

*Note:* Reference: Patients without CKD. Significance level: *p < 0.05, **p < 0.01, and ***p < 0.001. Multivariable OR/IRR was adjusted for patient age, ethnicity, Charlson's Comorbidity Index (adjusted), surgical approach, pelvic lymph node dissection, year of surgery, and hospital size.

Abbreviations: CI = confidence interval, CKD = chronic kidney disease, IRR = incidence rate ratio, LOS = length of stay, OR = odds ratio.

^a^ Exceeding the third quartile for the cohort.

### 3.3 Severity of Chronic Kidney Disease Versus Adverse In‐Hospital Outcomes

In additional analyses addressing adverse in‐hospital outcomes in relation to CKD severity and after full multivariable adjustment, a dose–response relationship was observed for seven of 14 comparisons (Table 3). Specifically, a step‐by‐step increase in relation to the severity of CKD was observed for overall complications (multivariable odds ratios for mild, moderate, and severe CKD: 1.83, 2.04, and 2.41), critical care therapies (ORs: 1.86, 2.95, and 3.37), dialysis rates for acute kidney failure (ORs: 2.95, 10.25, and 42.77), blood transfusions (ORs: 1.66, 1.72, and 3.69), cardiac complications (ORs: not‐significant, 1.92, 1.97), LOS > 2 days (ORs: 1.55, 1.78, and 2.77), and THC (multivariable IRR for mild, moderate, and severe CKD: 1.13, 1.21, and 1.25).

## 4 Discussion

RP patients with CKD represent a unique population at risk for adverse in‐hospital outcomes. We hypothesized that CKD patients may no longer be at a significant disadvantage when adverse in‐hospital outcomes after RP are considered. We tested this hypothesis and found several noteworthy results.

First, 4349 of 191 050 RP patients (2.3%) had CKD. Of those, 2.048 patients (47.1%) were classified as moderate or severe CKD. This cohort represents the largest contemporary repository of RP patients with CKD of all stages, which also included a relevant proportion of advanced CKD stages. Only two previous large‐scale studies specifically focused on complications in CKD patients undergoing RP [4, 5]. Schmid et al. reported on the effect of CKD on perioperative outcomes in major urological cancer surgeries, which also included 678 RP patients with moderate or severe CKD [4]. Ning et al. reported 1766 CKD and 273 end‐stage renal disease patients who underwent RP [5]. Moreover, both studies were substantially more historical (Schmid et al.: 2005–2011; Ning et al.: 2005–2014) than the current one (2005–2019). The remaining studies addressing RP in CKD patients were either small, historical, or did not address adverse in‐hospital outcomes [15, 16, 17, 18].

Second, the proportion of RP patients with underlying CKD increased sharply from 0.3% in 2005 to 5.6% in 2019 (EAPC: + 15.3%, *p* < 0.001). This increasing trajectory was universal across all CKD stages. Specifically, the relative increase was larger for advanced CKD stages (EAPC for moderate: +22.5%; EAPC for severe: +12.9%) than for mild CKD (EAPC: +11.4%, all *p* < 0.001). We are the first to report the rising importance of advanced CKD in surgically managed localized PCa. This validates the clinical relevance of the current study and the necessity of including more contemporary patients, who harbor more frequently advanced CKD stages, in contrast to previous reports [4, 5].

Third, we identified important differences in patient, hospital, and surgery characteristics that distinguished CKD patients. Specifically, CKD RP patients were older (66 vs. 62 years), more frequently African‐American (25.9% vs. 10.8%), exhibited a higher rate of CCI ≥ 2 (33.9% vs. 5.9%), more frequently underwent robot‐assisted RP (64.2% vs. 53.6%), and more frequently underwent concomitant PLND (50.8% vs. 46.8%). While the exact tumor stage is not available within the NIS, the increased PLND rate may be reflective of adverse cancer characteristics at RP in CKD patients. In accordance with the observed increase in CKD rates over time, a larger proportion of CKD patients underwent RP in more recent years (median, 2015 vs. 2011), and it may be expected that this proportion will continue to increase in the future. This temporal trend may also partly explain the higher utilization of the robot‐assisted approach in CKD patients. CKD patients were more frequently treated at small‐/medium‐sized hospitals (37.5% vs. 34.8%), which may be reflective of a more permissive practice in allowing more CKD patients to undergo RP. These characteristics (age, ethnicity, CCI, robot‐assisted approach, PLND, and smaller hospital) represent established predictors of in‐hospital outcomes [1, 19, 20, 21, 22]. Consequently, PSM and additional detailed multivariable adjustment for these recorded differences are required when such population differences distinguish cases (patients with CKD) from controls (patients without CKD). Such adjustments were universally applied in the current analysis. No previous study that focused on CKD patients undergoing RP used this technique for the end point of adverse in‐hospital outcomes [4, 5, 15, 16, 17]. As a consequence, significant biases and confounders may have been left unaddressed in previous studies, unlike in the current analysis.

Fourth, we addressed adverse in‐hospital outcomes of RP patients according to the presence or absence of CKD. After PSM, CKD was invariably associated with higher rates of adverse in‐hospital outcomes in all examined categories, except in‐hospital mortality. The absolute differences were largest for overall complications (+12.5%), length of stay > 2 days (+11.8%), blood transfusions (+3.7%), genitourinary complications (+3.0%), and cardiac complications (+2.8%). After additional multivariable adjustment, CKD was found to be an independent predictor of all adverse in‐hospital outcomes, except in‐hospital mortality. The detrimental effect was of largest magnitude for dialyses for acute kidney failure (OR 10.49), genitourinary complications (OR 2.47), critical care therapies (OR 2.45), infectious complications (OR 2.01), overall complications (OR 1.98), and blood transfusions (OR 1.95). Due to its rarity, our study was likely underpowered to detect differences in in‐hospital mortality. These findings provide robust proof that CKD continues to exert a strong detrimental effect on adverse in‐hospital outcomes after RP. Relying on a more historic and smaller cohort, Ning et al. reported a similar detrimental effect of CKD on postoperative complications (OR 1.36), urinary complications (OR 5.16), and LOS after RP [5]. Due to missing PSM and different definitions of adverse in‐hospital outcomes, further comparisons were hindered. Our results are also in general accordance with those of Schmid et al., who assessed adverse in‐hospital outcomes in CKD patients undergoing any major urological cancer surgeries, including but not limited to RP [4].

Finally, a dose–response effect after PSM and multivariable adjustment was reported in seven of 14 comparisons, where a more severe CKD stage was associated with a larger detrimental effect on adverse in‐hospital outcomes. For example, the relative increases in the overall complications were +83%, +104%, and +141% for mild, moderate, and severe CKD, respectively. Moreover, a step‐by‐step increase in relation to the severity of CKD was also observed for critical care therapies (+86%, +195%, and 237%), dialysis rates for acute kidney failure (+195%, +925%, and +4177%), blood transfusions (+66%, +72%, and +269%), cardiac complications (+21%, +92%, and +97%), LOS > 2 days (+55%, +78%, and +177%), and THC (+13%, +21%, and +25%). In contrast, only mild CKD was associated with an increased risk of vascular complications (+67%), whereas moderate or severe/end‐stage CKD was not. Although CKD itself increases the risk of vascular events such as venous thromboembolism (VTE) [23], the higher plasma drug concentration and higher pharmacological action of standard VTE prophylaxis due to reduced renal clearance may counterbalance this effect in advanced CKD. Moreover, the overall low event rate of some complication categories, such as wound complications, limited our ability to detect statistically significant findings in the subgroup analyses. No previous report has provided such a granularity of data. However, our results are consistent with those of Ning et al., who reported higher rates of postoperative complications in end‐stage renal disease than in other CKD stages [5]. The importance of CKD at RP represents not an all‐or‐none phenomenon. But instead, CKD severity is proportional to its effect on adverse in‐hospital outcomes. Possibly, a continuously coded GFR rate could allow us to ascertain a stronger dose–response relationship. Indeed, Tollefson et al. reported a strong association between GFR prior to RP and overall mortality in mostly non‐CKD patients treated between 1990 and 2004 [17]. However, coding of CKD in NIS is provided using categorical data, where floor/ceiling effects may weaken the true relationship between kidney function and adverse in‐hospital outcomes.

Taken together, the current study provided the largest contemporary cohort of CKD patients undergoing RP. The CKD rate at RP sharply increased over the study period (from 0.3% in 2005 to 5.6% in 2019), with the largest relative increase in moderate and severe CKD. CKD was invariably associated with a more unfavorable in‐hospital outcome profile in both absolute and relative comparisons. These disadvantages were broad and included all examined categories except in‐hospital mortality. Its magnitude was the largest for dialysis, genitourinary complications, and critical care therapies. Moreover, the dose–response relationship between CKD severity and its detrimental effects on adverse in‐hospital outcomes validated the applied methodology. If RP is considered in patients with severe CKD, close collaboration between urologists and nephrologists is mandatory to mitigate the detrimental effect of CKD by improved perioperative management. However, mild or moderate CKD did not exert a prohibitive effect that would clearly preclude RP as a treatment option if indicated.

Although a large‐scale analysis necessitates the use of retrospective, population‐based databases, the present study has inherent limitations that rely on this approach. First, our study shares the limitations of all similar studies that were based on the NIS or SEER databases and relied on a retrospective data design. Because treatment alternatives for localized PCa exist, confounding by the indication for RP may be assumed. Second, data regarding functional and oncological outcomes, as well as complications after the initial hospital stay (such as readmission rates) were not available. Third, detailed laboratory values and urine output data prior to RP were not available. However, Grams et al. reported a high specificity of kidney function assessment derived from ICD‐9 and ICD‐10 codes of > 98%, compared with the actual clinical values [24]. Fourth, information on the cancer stage was not available, which might have influenced patient selection, disease management, and adverse in‐hospital outcomes. Fifth, the current analysis relied exclusively on patients treated in the United States. Therefore, the presented results may not be representative of medical practices in other healthcare systems.

## Author Contributions

**Fabian Falkenbach:** conceptualization, investigation, writing – original draft. **Natali Rodriguez Peñaranda:** writing – review and editing, data curation. **Mattia Longoni:** writing – review and editing, visualization. **Andrea Marmiroli:** writing – review and editing. **Quynh Chi Le:** writing – review and editing. **Calogero Catanzaro:** writing – review and editing. **Michele Nicolazzini:** writing – review and editing. **Zhe Tian:** methodology, software, formal analysis. **Jordan A. Goyal:** validation. **Stefano Puliatti:** writing – review and editing. **Riccardo Schiavina:** writing – review and editing. **Carlotta Palumbo:** writing – review and editing. **Gennaro Musi:** writing – review and editing. **Felix K. H. Chun:** writing – review and editing. **Alberto Briganti:** writing – review and editing. **Fred Saad:** writing – review and editing, supervision. **Shahrokh F. Shariat:** writing – review and editing. **Lars Budäus:** writing – review and editing. **Markus Graefen:** writing – review and editing. **Pierre I. Karakiewicz:** conceptualization, writing – original draft, supervision, resources, project administration, funding acquisition.

## Disclosure

Institutional review board: The institutional review board waived study‐specific ethics approval owing to the anonymous design of the population‐based dataset (NIS).

## Ethics Statement

The authors have nothing to report.

## Consent

The authors have nothing to report.

## Conflicts of Interest

The authors declare no conflicts of interest.

## Supporting information


**Table S1.** Descriptive characteristics of prostate cancer patients undergoing robot‐assisted radical prostatectomy, stratified according to presence or absence of chronic kidney disease, prior and after 1:3 propensity score matching (PSM).
**Table S2.** Adverse in‐hospital outcomes after robot‐assisted radical prostatectomy for localized prostate cancer, stratified according to presence or absence of chronic kidney disease after 1:3 propensity score matching. (*n* = 11 164).
**Table S3.** Multivariable regression models predicting adverse in‐hospital outcomes according to presence or absence of chronic kidney disease (overall, stage‐specific) at robot‐assisted radical prostatectomy, after 1:3 propensity score matching and adjustment for clustering at the hospital level using generalized estimation equation methodology (*n* = 11 164).
