# Supplementary material for: The Effect of Chronic Kidney Disease on Adverse In‐Hospital Outcomes at Radical Prostatectomy
Source: Int J Urol. 2025 Mar 14;32(6):710–7. doi: 10.1111/iju.70038 (PMC12146236; doi:10.1111/iju.70038)
Supplement: Supplementary file 1 — Table S1. Descriptive characteristics of prostate cancer patients undergoing robot‐assisted radical prostatectomy, stratified according to presence or absence of chronic kidney disease, prior and after 1:3 propensity score matching (PSM). Table S2. Adverse in‐hospital outcomes after robot‐assisted radical prostatectomy for localized prostate cancer, stratified according to presence or absence of chronic kidney disease after 1:3 propensity score matching. (n = 11 164). Table S3. Multivariable regression models predicting adverse in‐hospital outcomes according to presence or absence of chronic kidney disease (overall, stage‐specific) at robot‐assisted radical prostatectomy, after 1:3 propensity score matching and adjustment for clustering at the hospital level using generalized estimation equation methodology (n = 11 164). [file IJU-32-710-s001.pdf]

Table S1: Descriptive characteristics of prostate cancer patients undergoing robot-assisted radical prostatectomy, stratified according to presence or absence of chronic kidney disease, prior and after 1:3 propensity score matching (PSM).

	Prior PSM			After 1:3 PSM		
**Characteristic**	**With CKD** (n = 2,791, 2.7%)	**Without CKD** (n = 100,083, 97.3%)	*p-value*[1]	**With CKD** (n = 2,791, 25.0%)	**Without CKD** (n = 8,373, 75.0%)	*p-value*[1]
**Age**, median (IQR), in years	66 (60, 70)	62 (57, 67)	<0.001	66 (60, 70)	66 (61, 70)	0.8
**CKD stage,** n (%)			NA			NA
**mild (stage I/II)**	1,424 (51.0%)	0 (0%)		1,424 (51.0%)	0 (0%)	
**moderate (stage III)**	982 (35.2%)	0 (0%)		982 (35.2%)	0 (0%)	
**severe/end-stage (stage IV/V)**	385 (13.8%)	0 (0%)		385 (13.8%)	0 (0%)	
**Adjusted CCI**[2] **,** n (%)			<0.001			0.8
**0**	1,219 (43.7%)	74,120 (74.1%)		1,219 (43.7%)	3,707 (44.3%)	
**1**	651 (23.3%)	19,860 (19.8%)		651 (23.3%)	1,950 (23.3%)	
**≥2**	921 (33.0%)	6,103 (6.1%)		921 (33.0%)	2,716 (32.4%)	
**Ethnicity,** n (%)			<0.001			0.4
**Caucasians**	1,545 (55.4%)	69,031 (69.0%)		1,545 (55.4%)	4,747 (56.7%)	
**African-Americans**	737 (26.4%)	11,665 (11.7%)		737 (26.4%)	2,179 (26.0%)	
**Others**	509 (18.2%)	19,387 (19.4%)		509 (18.2%)	1,447 (17.3%)	
**PLND,** n (%)	1,564 (56.0%)	48,909 (48.9%)	<0.001	1,564 (56.0%)	4,699 (56.1%)	>0.9
**Year of surgery,** n (%)	2016 (2013, 2017)	2014 (2011, 2016)	<0.001	2016 (2013, 2017)	2016 (2013, 2017)	0.8
**Small-/medium-sized hospital,** n (%)	1,076 (38.6%)	38,713 (38.7%)	0.9	1,076 (38.6%)	3,270 (39.1%)	0.7

[1]**Wilcoxon rank-sum test, Pearson's chi-square test.** [2]**The point contribution to CCI due to CKD was subtracted.**

Abbreviations: CKD = chronic kidney disease, IQR = Interquartile range, PSM = propensity score matching, PLND = pelvic lymph node dissection.

PSM relied on patient age, ethnicity, Charlson Comorbidity Index (adjusted), PLND, year of surgery, and hospital size.

Table S2: Adverse in-hospital outcomes after robot-assisted radical prostatectomy for localized prostate cancer, stratified according to presence or absence of chronic kidney disease after 1:3 propensity score matching. (n= 11,164)

Characteristic	**With CKD** (n = 2,791, 25.0%)	**Without CKD** (n = 8,373, 75.0%)	Difference (Δ)	*p-value*[1]
**Overall complications,** n (%)	777 (27.8%)	1,423 (17.0%)	10.8%	<0.001
**Critical care therapies** (without dialysis)**,** n (%)	63 (2.3%)	74 (0.9%)	1.4%	<0.001
**Dialysis for acute kidney failure,** n (%)	17 (0.6%)	<11 (<0.1%)	>0.5%	<0.001
**Bleeding complications,** n (%)	31 (1.1%)	60 (0.7%)	0.4%	0.0596
**Blood transfusions,** n (%)	99 (3.5%)	136 (1.6%)	1.9%	<0.001
**Cardiac complications,** n (%)	221 (7.9%)	447 (5.3%)	2.6%	<0.001
**Respiratory complications,** n (%)	111 (4.0%)	140 (1.7%)	2.3%	<0.001
**Genitourinary complications,** n (%)	95 (3.4%)	110 (1.3%)	2.1%	<0.001
**Wound complications,** n (%)	<11 (<0.4%)	<11 (<0.1%)	>0.1	0.047
**Infectious complications,** n (%)	38 (1.4%)	33 (0.4%)	1.0%	<0.001
**Vascular complications,** n (%)	38 (1.4%)	61 (0.7%)	0.6%	0.003
**In-hospital mortality,** n (%)	<11 (<0.4%)	11 (0.1%)	>0.1%	0.3
**Prolonged LOS (>2 days)**[2], n (%)	751 (26.9%)	1,435 (17.1%)	9.8%	<0.001
**THC,** median (IQR), in US$	58,230 (40,210, 89,850)	52,240 (36,930, 77,440)	5,990 US$	<0.001

[1]Wilcoxon rank-sum test, Pearson's chi-square test. [2] Exceeding the third quartile for the cohort.
Abbreviations: CKD = chronic kidney disease, IQR = interquartile range, LOS = length of stay, THC = total hospital charges.

Table S3: Multivariable regression models predicting adverse in-hospital outcomes according to presence or absence of chronic kidney disease (overall, stage-specific) at robot-assisted radical prostatectomy, after 1:3 propensity score matching and adjustment for clustering at the hospital level using generalized estimation equation methodology. (n= 11,164)

	Multivariable OR/IRR (95% CI)			
**Characteristic**	**CKD** (all stages, n=2,791)	**mild CKD** (stage I/II, n=1,424)	**moderate CKD** (stage III, n=982)	**severe/end-stage CKD** (stage IV/V, n=385)
**Overall complications**	1.93 (1.73-2.14)***	1.78 (1.55-2.05)***	1.99 (1.71-2.32)***	2.34 (1.85-2.97)***
**Critical care therapies** (without dialysis)	2.59 (1.84-3.64)***	1.82 (1.14-2.90)*	3.57 (2.33-5.48)***	2.87 (1.44-5.72)**
**Dialysis for acute kidney failure**	10.43 (3.82-28.45)***	1.13 (0.13-9.75)	11.47 (3.42-38.46)***	45.51 (14.56-142.20)***
**Bleeding complications**	1.56 (1.01-2.41)*	1.84 (1.10-3.08)*	0.77 (0.31-1.90)	2.37 (1.09-5.15)*
**Blood transfusions**	2.24 (1.73-2.89)***	1.94 (1.39-2.71)***	1.59 (1.05-2.43)*	4.98 (3.31-7.50)***
**Cardiac complications**	1.56 (1.31-1.86)***	1.14 (0.88-1.48)	1.92 (1.51-2.43)***	2.22 (1.48-3.34)***
**Respiratory complications**	2.42 (1.89-3.11)***	2.07 (1.50-2.86)***	2.91 (2.09-4.07)***	2.40 (1.33-4.33)**
**Genitourinary complications**	2.65 (2.00-3.52)***	2.78 (1.98-3.91)***	2.75 (1.86-4.07)***	1.95 (1.01-3.77)*
**Wound complications**	2.76 (1.11-6.86)*	3.00 (1.02-8.81)*	1.81 (0.38-8.69)	4.07 (0.86-19.38)
**Infectious complications**	3.45 (2.14-5.54)***	2.27 (1.17-4.41)*	4.87 (2.73-8.68)***	3.51 (1.29-9.52)*
**Vascular complications**	1.90 (1.26-2.86)**	1.91 (1.13-3.25)*	1.92 (1.10-3.37)*	1.75 (0.49-6.22)
**In-hospital mortality**	1.84 (0.70-4.86)	1.73 (0.48-6.21)	1.89 (0.50-7.11)	2.08 (0.24-17.80)
**Prolonged LOS (>2 days)†**	1.76 (1.59-1.95)***	1.50 (1.31-1.71)***	1.74 (1.50-2.02)***	3.09 (2.48-3.85)***
**Total hospital charges**	1.14 (1.11-1.18)***	1.09 (1.06-1.12)**	1.18 (1.11-1.25)**	1.26 (1.16-1.37)**

Reference: patients without CKD. Significance level: * p<0.05, **p<0.01, *** p<0.001. Multivariable OR/IRR was adjusted for patient age, ethnicity, Charlson Comorbidity Index (adjusted), pelvic lymph node dissection, year of surgery, and hospital size. † Exceeding the third quartile for the cohort.
Abbreviations: CKD = chronic kidney disease, OR = odds ratio, IRR = incidence rate ratio, CI = confidence interval, LOS = length of stay.
